# Dorsal root entry zone lesion: nuances of the technique and long-term results

**DOI:** 10.3171/2020.7.FOCVID2031

**Published:** 2020-10-01

**Authors:** Stefano Ferraresi, Elisabetta Basso, Lorenzo Maistrello, Alba Scerrati, Piero Di Pasquale

**Affiliations:** Department of Neurosurgery, Ospedale Santa Maria della Misericordia, Rovigo, Italy

**Keywords:** dorsal root entry zone lesion, DREZ, brachial plexus, lumbosacral plexus, root avulsion

## Abstract

The treatment of deafferentation pain is a primary goal of a referral center for peripheral nerve surgery. DREZ is an important asset in the neurosurgeon’s armamentarium. The surgical technique and long-term results are analyzed in two series, with or without intraoperative monitoring (IOM). DREZotomy is highly effective in lumbar root avulsive injuries but is ineffective in resolving pain due to spinal cord injuries. Cervical DREZotomy for cancer pain is not superior to intrathecal morphine. In brachial plexus avulsive injuries, the largest series shows a 74% success rate, but the efficacy of the procedure is lost over time. No relevant difference has been observed since the introduction of IOM.

The video can be found here: https://youtu.be/uG_kkQj5m1U

## Transcript

This video illustrates our experience with microDREZotomy, by far the most effective procedure in the treatment of deafferentation pain due to avulsive injuries of the lumbar and brachial plexuses.

**0:59** Our data share similar results with other authors.^1–5^ The dorsal DREZotomy, devised for pain due to traumatic spinal cord injury, is no longer performed because it is ineffective. The best results are obtained with the lumbar DREZ for monolateral or bilateral avulsive injury of the lower lumbar roots, namely L5 and S1. The cervical DREZotomy also plays a big role in the treatment of neuropathic pain due to avulsive injuries of the brachial plexus. Cancer infiltration of the cervical roots may be effective, but the results are far more unpredictable. Our series (1997 to current date) has been divided into two groups, before and after the year 2007. In fact, since 2007 we have had at our disposal neurophysiological intraoperative monitoring during brain and spinal surgery.

The oldest group is more numerous because nowadays, in the meanwhile, a wide array of new drugs has appeared, some of them being able to soothe the excruciating neuropathic pain so that many patients prefer resorting to a conservative approach instead of undergoing a rather invasive surgery.

The video is focused on the cervical microDREZotomy for brachial plexus avulsive injuries. The most numerous group is made of total palsies; therefore, the vast majority of the patients underwent a standard complete cervical DREZotomy.

**1:10** The two other groups, namely the one with C5–C6–C7 roots avulsed and the avulsive injuries of the lower roots, are less represented. In panavulsive injuries, the pain is mainly referred to the arm, forearm, and to the hand, mostly on the ulnar side, although some patients occasionally refer to the thumb as more painful than the fourth and fifth fingers. Upper root injuries characteristically experience neuropathic pain in the dorsoradial area of the thumb and index finger, particularly corresponding to the very distal phalanx of the two fingers.

The pain is perceived as a burning sensation, a continuous stabbing, sudden discharges of electricity, the biting of a dog, the hand crushed like in a vice, and, occasionally, but very peculiarly, as a phantom hand placed in the back.

The goal of the surgery in the DREZ is the destruction of the interneurons in the substantia gelatinosa, deep in the gray of the dorsal horn. These neurons, suddenly deprived of the proprioceptive afferent signals via the roots, also including a modulating inhibitory function, “feel free” to discharge without control, short-circuiting the normal pathways to the thalamus and the sensory cortex. The aim of the surgery, through heat due to microcoagulation, radiofrequency, or ultrasound, is to destroy these “sick” interneurons, so stopping the continuous impulses arriving to the brain.

The approach to the dorsal root entry zone is ipsilateral to the side of plexus injury, through the ipsilateral posterolateral sulcus of the cervical (or lumbar) spinal cord with an angle approximately of 45°, paying attention to stay anterior to the dorsal rootlets, when some of them are left, directing the tip of the bipolar into a depth from 3 to 5 mm, into the gray matter, and trying to accurately separate the cords as it happens when squeezing longitudinally a banana.

Note how the area of the DREZ is immediately lateral to the pyramidal tract, in the sector of the lower limb. One can easily understand how a mistake in placing the neurolesion may cause a loss of strength in the leg ipsilateral to the affected arm. A mistake toward the opposite side of the DREZ, in turn, can entail thoracic dysesthesias and a loss of proprioception again in the ipsilateral lower limb.

**2:00** The basics of surgery are here illustrated, and the mainstays of the procedure are listed as following.

**2:44** Positioning. The patient is placed in prone position with a Mayfield headset, with a silicon pad under the chest and stiff pads under the iliac crests, to be sure that no movement occurs during this very delicate procedure and particularly during the elicitation of intraoperative motor evoked potentials. Crisscross shoulder straps are used to stretch down the shoulders and expose as many cervical segments as possible without the lateral interference of the scapulae. An x-ray image intensifier is brought into the surgical setting to identify the exact extent of the hemilaminectomy. The surgical bed is tilted like a sink to minimize the sliding of the patient forward and backward, and with the head slightly flexed to simulate a Concorde position. This is to minimize the descent of CSF from the cranial compartment, with resulting pneumocephalus.

**3:16 Bone Exposure of the Cervical Spine.** The exposure is rigorously obtained via an hemilaminectomy variably including the laminae from C4 to T2. We are very cautious not to trespass the facet joint and work only on the laminae. This ensures no long-term problems in stability of the spine.

Also to increase stability of the cervical complex and diminishing the neck pain due to the approach, we always do our best to preserve a tiny rim of the upper border of C4 (in case it is the more proximal lamina) and a tiny rim of bone in the distal part of T2 (or in the last lamina to be uncovered).

The extension of the hemilaminectomy depends upon the type of the required microDREZotomy and rests entirely upon clinical criteria. The distribution of the deafferentation pain, and *not* the preoperative studies, dictates the type of microDREZotomy.

Nevertheless, we usually perform a 3D CISS MR myelography of the brachial plexus^6^ (in the older cases, a myelogram) and, if the patients have been operated elsewhere for nerve reconstruction, we always require the surgical report. This allows us to review the results obtained by the first surgery: sometimes an anatomically normal root which has proved not to be functioning may contribute to the generation of pain and is better included in the DREZ lesion. As a rule of the thumb, in total brachial plexus palsies, even though pain is usually referred in the forearm and hand, we perform a complete C4 to T2 hemilaminectomy and a full C5–T1 microDREZotomy, as illustrated in the video example.

This is because, as we will see later on, part of the pain located in the hand may arise from the avulsive injury of the C5 and C6 roots.

In lower brachial plexus injuries (so-called pure Dejerine-Klumpke from the onset), the upper limit of the hemilaminectomy can be set to C6 and a reduced C6–T2 exposure is enough. In pure upper C5–6 avulsive injuries (so-called Erb-Duchenne from the onset), which very rarely require a DREZotomy to treat the neuropathic pain radiating to the dorsal thumb and index finger, the lower limit can be halted in T1. In this latter group of injuries, we prefer to include the lamina of C7 in the bone removal, to be able to easily reach the root C8, which is, not rarely, the first normal root (avulsive injuries of C5–6 and C5–C6–C7 can share the same clinical presentation). The upper and lower limit of the microDREZotomy in these latter cases are the sound roots.

**4:03 Intradural Technique.** Before opening the dura, the electrode of the D-wave is placed extradurally and, once verified, is secured to the muscle with stitches and to the skin with an adhesive drape. D-wave is a very important part of the neuromonitoring because a significant reduction of 50% of this wave during surgery forces the surgeon to stop the procedure, because it indicates the possibility of a permanent neurological damage.

Once the dura is opened, we check the anatomy of the roots to define which are avulsed and which are not. Occasionally, some residual dorsal rootlet can be still in place, in spite of the total avulsion of the ventral rootlets. If it is the case, this is a valuable help to identify the posterolateral sulcus and the DREZ (the zone of entry of the dorsal rootlets). In total avulsive injuries, one can seek help in the nearby normal roots or make a visual judgment if there is not much scar on the cord, or can choose to rest entirely on the intraoperative monitoring, or the combination of all these assets.

After the introduction of the IOM, we always make a check and await confirmation from the electrical stimulation. The posterolateral sulcus and the DREZ area should be neutral in terms of neural conduction, and the response wave should remain flat or almost flat. Once the stimulation shifts toward the pyramidal tract, we observe a positive wave, which becomes higher in the center of the pyramidal tract. Conversely, moving to the spinocerebellar cords, a wave pointing to the opposite direction is elicited. Do not stick with the idea of the positive or negative spikes because it is an arbitrary setting; what we need to know is that the DREZ area should be neutral (flat signal), while as soon as we move to the motor and sensory “noble” areas, spikes of opposite signs are elicited.

### DREZ Stimulation Parameters

Recording: two-lead strip located subdurally on the spinal cord distally to the region of interest (not averaged responses—filters 20 Hz to 5 kHz).

Stimulation: bipolar probe, intensity 0.1–0.5 mA; distance between probe and recording leads from 3 to 5 cm.

Once the posterolateral sulcus is identified beyond any doubt, a very superficial coagulation of the tiny vessels of the sulcus is a necessary step before cutting the arachnoid with a microscalpel or an ocular lancet. Then we follow a 45° angle using a dissector and the bipolar with the intent of separating the cords like a banana.

The easier this process appears, the more likely we are able to perfectly reach the gray matter of the dorsal horn in the substantia gelatinosa and perform a low-intensity coagulation to a minimal depth of 3 up to 5 mm. The tip of the bipolar has been prepared with millimeter marks to help the surgeon and stop once the correct depth is reached.

Sometimes, to assist the sulcus in staying spread apart, we insert a very thin strip of Spongostan in the depth of the sulcus, without consequences.

**5:14 Important Tip.** The angle of the bipolar should be changed approaching T1 because the DREZ area tilts vertical and steeper. The risk is, obviously, to trespass the pyramidal or the spinocerebellar tracts.

Pay attention not to violate noble tracts either dorsally or ventrally.

The dura is then closed watertight with 4-0 silk stitches and apposition of T-dural sealing sheet.

**5:36** Postoperative MRI Showing the DREZ Lesion on the Cord

The lumbar DREZotomy^7^ follows the same principles. A hemilaminectomy (monolateral pain) or a bilateral T10–L2 laminectomy (bilateral pain) widely exposes the area of the conus medullaris.

The monitoring of the anal sphincter is mandatory since the most affected painful areas usually are those pertaining to the foot (or feet) and namely S1, which is located medially in the conus, near the “sphincteric” neuromeres S2 and S3.

For this reason, it is important to monitor the function of the anal sphincter, especially in the bilateral cases. This is a very simple and easy setting, but avoids a very annoying complication in the short and long term. The rest of the procedure does not substantially differ from the cervical DREZ. The results are usually very satisfactory. In terms of percentage of success, they constitute the best score in our series.

**5:45 Short Summary of the Surgical Technique of Lumbar DREZotomy.** The results are more or less the same before and after the introduction of intraoperative monitoring: no significant difference between the two groups as to the number and type of complications.

**6:33** This is the most typical complication: the weakness of the ipsilateral leg is due to a pyramidal tract manipulation, but, more often, it takes the appearance of a deficit of the proprioceptive type. Characteristically, the weakness improves by having the patient stare at his/her legs and feet.

Thoracic dysesthesias are the second most typical problem encountered after surgery. They do not entail a very significant deficit, but many patients refer to them as very annoying and probably depend on the violation, even for a short territory, of the spinocerebellar tracts. The position is immediately dorsolateral to the dorsal horn.

They tend to disappear with time, but this is unpredictable. We have several patients still experiencing this exquisitely subjective problem after many years.

### Short- and Long-Term Results

**6:57** Immediately after surgery, we have a good rate of success (74%), but still remarkable is the high rate of failures, if one thinks that we are dealing with a very delicate and invasive surgery and that the patients, most of the time, have waited for a long time before surgery and with strong expectations due to the very severe pain.

As a matter of fact, if we put together the failures and the complications, for various reasons we will have 40% of patients who are unhappy immediately after surgery, and this must be a warning to be given during the informed consent.

Moreover, we have not completely understood all the factors leading to an unsuccessful surgery. The technique is the same and the surgeon is the same and, apart from the skipping of areas corresponding to sound roots, as we will see later, we have no explanations at hand.

**7:30** The long-term results are nicely illustrated through multiple graphs showing the loss of the antalgic effect with time.^8–10^

If 75% of the patients have a good antalgic effect immediately after surgery, this score tends to fade away over the years. At a 5-year follow-up, about half of the patients originally free from pain have developed a lighter, albeit dull, aching, which is no longer a devastating experience but still leaves them not completely satisfied. For this reason, differently from what we did in the past, we have learned to administer half the quantity of analgesic drugs, without stopping them abruptly as we did in the past.

A not negligible percentage, then, experience an unexpected return of severe pain (13%).

The return of an unbearable pain is a big problem, since after the very first month after surgery a thick scar develops in the cord, and recognition of the posterolateral sulcus may be impossible, even with electrode stimulation and recording, as we witnessed on one occasion.

Under select circumstances, the repetition of the DREZ procedure is possible, in the early postoperative stage, and usually consists of an extension of the procedure more upward or downward, toward painful levels of neuromeres not covered by the first surgery.

The repetition into the depth of an already operated posterolateral sulcus is, on the contrary, much more risky and should not be challenged, especially if it occurs years after the first procedure.

### Tips and Tricks

**8:05** Very important: generally speaking, the patients having four- or five-root avulsive injuries score better.

A fully extended exposure from C4 to T2 with good exposure of the distal neuromeres and the sulcus corresponding to the areas of the roots C8 and especially T1 is likely to give the most successful results.

At times, even in total palsies, some root may be anatomically preserved, and one is tempted to skip the corresponding area of the DREZ for fear of damaging some neurological function, if preserved. We strongly warn against this practice: our experience has clearly shown that this is associated with a higher rate of failure in relieving pain. The corresponding gain in sensory loss, on the contrary, is negligible.

Therefore, remember that in case of preservation of a normal root between two avulsed roots, the line of the DREZ surgery must be uninterrupted.

Do not skip apparently sound neuromeres—they are not normal anyway.

These are the most important tricks to minimize failures, which, to some extent, remain unexplained in many patients.

This has probably to do with the empirical basis of the procedure, and there must be other factors that we are not able to account for that are responsible for the failures.

The time interval between onset of pain and DREZ procedure doesn’t seem to be correlated with the success rate. We’ve had excellent results in very late cases and impressive failures in recently injured patients.

Is there any solution for neuropathic pain unresponsive to DREZotomy? The only possibility to cure is left to the motor cortex stimulation, the results of which are also very unpredictable. From the scanty reports we have, it does not cover more than 30%–40% of the remaining cases.

## Author Contributions

Primary surgeon: Ferraresi. Assistant surgeon: Basso, Maistrello. Editing and drafting the video and abstract: Ferraresi, Basso. Critically revising the work: Ferraresi, Scerrati. Reviewed submitted version of the work: Ferraresi, Scerrati. Approved the final version of the work on behalf of all authors: Ferraresi. Supervision: Ferraresi, Maistrello, Scerrati. Anesthesiologist and neurophysiologist: Di Pasquale.

## Supplemental Information

### Previous Presentations

This abstract and operative video demonstration were presented in part at the 44th Congress of the Romanian Society of Neurosurgery, Timisoara, Romania, September 5–8, 2018.
